# Influence of Dietary Intake on Carotid Maximum Intima–Media Thickness in Children Conceived Through Assisted Reproductive Techniques

**DOI:** 10.3390/nu17071189

**Published:** 2025-03-28

**Authors:** Blanca Barrau-Martinez, Mireia Termes-Escalé, Brenda Valenzuela-Alcaraz, Rafael Llorach, Andreu Farran-Codina, Alba Tor-Roca, Eduard Gratacós, Fatima Crispi, Mireia Urpi-Sarda

**Affiliations:** 1 Departament de Nutrició, Ciències de l’Alimentació i Gastronomia, Facultat de Farmàcia i Ciències de l’Alimentació, Campus de l’Alimentació de Torribera, Universitat de Barcelona (UB), 08921 Santa Coloma de Gramenet, Spain; blancabarrau@ub.edu (B.B.-M.);; 2 Institut de Recerca en Nutrició i Seguretat Alimentària (INSA-UB), Campus de l’Alimentació de Torribera, Universitat de Barcelona (UB), 08921 Santa Coloma de Gramenet, Spain; 3 Centre de Medicina Maternofetal i Neonatal de Barcelona (BCNatal), Hospital Clínic and Hospital Sant Joan de Déu, IDIBAPS, 08028 Barcelona, Spain; 4 Centro de Investigación Biomédica en Red de Fragilidad y Envejecimiento Saludable (CIBERFES), Instituto de Salud Carlos III, 28029 Madrid, Spain; 5 Centro de Investigación Biomédica en Red de Enfermedades Raras (CIBERER), Instituto de Salud Carlos III, 28029 Madrid, Spain

**Keywords:** assisted reproduction technologies, food intake, nutrition, dietary patterns, carotid maximum intima–media thickness, cardiovascular programming, vascular remodeling, nutritional recommendations, saturated fatty acids

## Abstract

**Background/Objectives**: Research on the relationship between nutritional characteristics and their impact on cardiovascular remodeling in children conceived by assisted reproductive technology (ART) is limited. We aimed to explore the influence of postnatal nutrition on vascular wall thickness in children conceived through ART, comparing them with a naturally conceived control group. **Methods**: A prospective observational cohort of 3-year-old children (n = 83) was analyzed, including 41 conceived ART and 42 spontaneously conceived. The carotid maximum intima–media thickness (max-cIMT), a strong predictor of myocardial infarction, was measured and dietary intake was assessed through 3-day food records. Dietary data were compared between groups, and the relationship between nutritional intake and max-cIMT were explored. In the ART group, the k-means clustering method identified distinct dietary patterns. **Results**: ART children showed significantly higher max-cIMT values, as well as increased dietary intake of saturated fatty acids (SFA), total proteins, and animal proteins compared to those spontaneously conceived. Three cluster groups were identified based on dietary intake in the ART group; those ART children whose dietary pattern closely resembled the control group exhibited lower max-cIMT values. **Conclusions**: Our findings suggest that ART children exhibited a distinct dietary pattern characterized by higher consumption of total and animal proteins and SFA, compared to those conceived naturally. Further research is required to unravel the interindividual differences among individuals conceived through ART, enabling the formulation of precise nutritional recommendations for personalized nutrition and preventive medicine.

## 1. Introduction

Assisted reproductive technologies (ARTs) have witnessed a global surge, contributing to 1–5% of all newborns, with an annual application rate exceeding 1 million worldwide [1,2]. Accumulating evidence suggests potential adverse effects of ART on the health of individuals conceived through these methods and several concerns have been raised regarding their long-term cardiovascular and metabolic well-being [3]. Previous data on cardiometabolic effects in children and adolescents conceived through ART indicate less favorable cardiovascular metabolic profiles [4], increased peripheral adiposity [5], vascular dysfunction [6], elevated blood pressure [3], and fasting glucose [7].

Cardiovascular programming, a phenomenon with implications at various life stages, has been observed in children conceived through ART [8], and it is manifested by cardiac and vascular remodeling persisting from fetal development into postnatal life [9]. Vascular remodeling, assessed through carotid intima–media thickness (cIMT), serves as a reliable indicator of early-stage artery hardening (atherosclerosis) and is pivotal in evaluating subclinical atherosclerosis [10]. Specifically, carotid maximum intima–media thickness (max-cIMT) is a stronger predictor of myocardial infarction than carotid mean intima–media thickness [cIMT (mean)], as it may more accurately reflect localized atherosclerotic plaques [11].

Among modifiable risk factors for cardiovascular disease (CVD), the role of diet and healthy eating is well-established. Previous prospective observational cohort studies suggested that unhealthy childhood nutrition (i.e., a diet poor in fruits and vegetables) can be linked to adult CVD risk, to early vascular changes predictive of CVD, and to elevated adulthood cIMT [12]. Understanding the influence of dietary intake on cardiovascular outcomes is crucial, given the emerging evidence on cardiovascular programming in various population groups (i.e., restricted intrauterine growth, obesity, hypercholesterolemia), and the mechanism through which nutrition influences vascular health [13]. Despite this, the association between nutrition and cardiac remodeling in individuals conceived through ART remains unexplored. Therefore, this study aimed to investigate the influence of children’s diet at 3 years old on max-cIMT in children conceived through ART, compared with a naturally conceived control group, addressing a critical gap in existing research on nutrition and cardiovascular health in the ART-conceived population.

## 2. Materials and Methods

### 2.1. Participants and Study Population

The comprehensive prospective cohort study involving children conceived using ART (n = 80) and spontaneously conceived (n = 80) recruited from fetal life and followed up to the age of 3 years, has been previously published elsewhere [8,9]. Briefly, exclusion criteria were preimplantation genetic diagnosis, oocyte donation, maternal comorbidities (such as diabetes, lupus, HIV or hepatitis C infection, kidney disease, etc.), twin monochorionic gestations, chromosomal anomalies, fetal malformations, or fetal infections and very preterm birth (e.g., delivery before 34 completed weeks of pregnancy).

**Figure 1:**
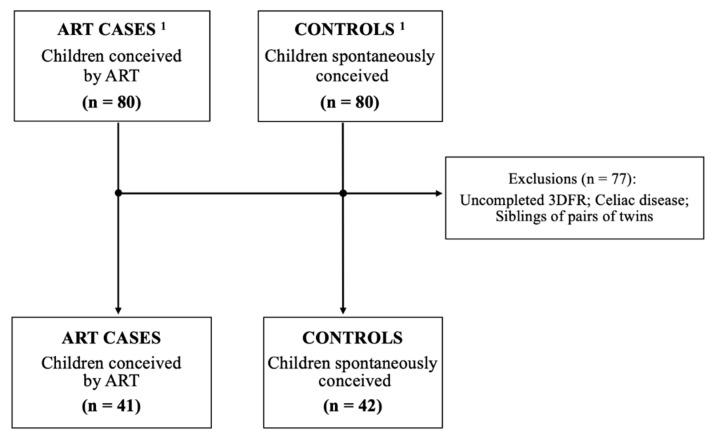
Flow chart of inclusion of participants in the study ^1^. Cohort described in previous studies of Valenzuela-Alcaraz et al. [8,9]. ART, assisted reproductive technology; 3DFR, three-day food record.

The current secondary analysis used characteristics gathered from 83 children (41 children conceived through ART and 42 naturally conceived) whose dietary information, obtained through a 3-day food record (3DFR), was completed. This was conducted in conjunction with cardiovascular assessments, the collection of anthropometric data, and various surveys. In instances involving twin participants, one subject from each pair was randomly excluded when intake data were highly similar between the twins or if a pair of siblings provided only a single 3DFR (Figure 1).

The project was carried out in compliance with the Hospital Clinic Barcelona Ethics Committee. The study protocol received approval from the Institutional Review Board at Hospital Clinic (HCB/2011/6247), and written informed consent was obtained from the parents of all study participants.

### 2.2. Assessment of Dietary Intake

In the current study, dietary intake was assessed using a standardized 3DFR over three consecutive days. Parents weighed and recorded all foods and beverages consumed by their children. The 3DFR data were coded and analyzed by trained dietitians using the nutritional evaluation software program PCN Pro version 1.32 (Barcelona, Spain) [14] to calculate total energy and nutrient intakes of the participants. Dietary intake was compared to recommended amounts from Recommended Dietary Allowances (RDA) [15].

Energy, macronutrients, and sodium intakes were expressed as kcal/kg/day, g/kg/day and mg/kg/day, respectively. These expressions align with typical recommendations for children, considering the intervariability, which is a determinant factor in calculating individual requirements for both energy and protein [16].

### 2.3. Vascular Evaluation

Vascular assessment included carotid wall thickness using ultrasound and blood pressure at 3 years old during the medical evaluation of participants.

Carotid ultrasound was conducted by a trained sonographer using a Vivid q system from General Electric Healthcare (Horten, Norway), as previously described [8]. Briefly, longitudinal clips of the far walls of both carotid arteries were obtained approximately 1 cm proximal to the bifurcation using a 3.33–10.0 MHz linear-array transducer. The carotid maximum IMT (max-cIMT) was subsequently measured offline according to a standardized protocol based on a trace method, with the assistance of the computerized program Echopac Software Only (Milwaukee, WI, USA). To obtain max-cIMT, three end-diastolic still frames were selected across a length of 10 mm and analyzed for maximum IMT [17].

Systolic and diastolic blood pressures were obtained from the brachial artery using a validated ambulatory automated Omron 5 Series device (Koto, Japan), while the child was resting. Mean blood pressure was calculated as ((2 × diastolic) + systolic)/3.

### 2.4. Other Variables

The characteristics of participants and the perinatal data were obtained at baseline: sex, gestational age at delivery, birth weight, birth height, and duration of breastfeeding. At 3 years of age, collected data included age, weight, height, and body mass index (BMI). The percentile values for weight, height, length, and BMI were calculated using the Intergrowth-21 growth curves [18] for perinatal data and the World Health Organization (WHO) growth curves for children’s data [19].

### 2.5. Statistical Analysis

The characteristics and perinatal data of the participants along with dietary information, were expressed as mean values and standard deviations (SD). Categorical variables were presented as numbers and percentages. The Kolmogorov–Smirnov and Levene’s tests were carried out to assess data distribution and homogeneity of variance. Variables with a skewed distribution were transformed into their natural logarithm for analyses. To compare characteristics and dietary intake between the groups, we used Student's *t*-test, the chi-square test or Fisher’s Exact test when appropriate. We used the *t*-test to evaluate the differences between the dietary intake of children in this cohort and the recommended nutritional guidelines [15]. In addition, differences between groups were analyzed by one-way ANCOVA (analysis of covariance) adjusted for potential cofounders (gestational age at delivery, birth weight, and birth length).

The relationship between max-cIMT and nutrient intake was evaluated using Pearson correlation coefficients in all participants and considering the group. When significant correlations were observed, multivariate linear regression analyses were performed to study the association between max-cIMT and nutrient intake and adjusting for the potential cofounders mentioned before. Effect modification by group was assessed by adding product terms in the fully adjusted regression models. Bonferroni correction was utilized for multiple testing correction (*p*-value < 0.05/14, statistical threshold at α = 0.004). Additionally, a statistical power analysis was conducted for the significant results [20].

Cluster analysis for identifying dietary patterns in the ART group was performed through the k-means cluster algorithm in MetaboAnalyst 5.0 [21]. This process yielded three distinct clusters among the ART participants, utilizing the dataset that encompassed energy and macronutrient intakes as input. Differences in the nutritional profiles between the spontaneously conceived and the three ART clusters were analyzed employing one-way ANOVA (analysis of variance) and ANCOVA with the Bonferroni post hoc test adjusted for potential confounders (gestational age at delivery, sex, weight, and height).

Statistical analyses were carried out using SPSS software package (SPSS 27.0, IBM, Armonk, NY, USA) and the significance level was maintained at *p*-value < 0.05.

## 3. Results

### 3.1. Characteristics of the Study Participants

Perinatal data were similar between the ART and spontaneously conceived groups (Table 1). At the time of evaluation, the mean ± SD age of the participants was 3.2 ± 0.6 years in the control group and 3.0 ± 0.5 years in the ART group, with no significant differences noted. As expected, the ART group had significantly lower birth length and gestational age at delivery compared to those spontaneously conceived. The max-cIMT value was significantly higher in the ART children (*p*-value < 0.001).

### 3.2. Evaluation of Child’s Nutritional Characteristics

ART children showed significantly higher consumption of total protein, animal protein, and saturated fatty acids (SFA) compared to those spontaneously conceived (adjusted *p*-value < 0.05) (Table 2). Moreover, children in the ART group showed a tendency towards higher sugar and total lipid intake compared to the control group (*p*-value = 0.07). It is worth noting that compared with Recommended Dietary Allowances (RDA) [15], total protein and total lipids intakes significantly exceeded the recommended values in both the ART and control groups (*p*-value < 0.001). However, total energy intake and total carbohydrates fell short of the recommended values in both groups (*p*-value < 0.001) (Supplementary Table S1).

### 3.3. Association Between Child’s Dietary Intake and Carotid Maximum Intima–Media Thickness

Correlation analysis demonstrated a significant correlation between the intake of SFA and sodium with max-cIMT (r = 0.249, *p*-value = 0.023; r = 0.256, *p*-value = 0.019, respectively) in the entire participant sample (n = 83) (Supplementary Table S2). A non-significant trend was observed between max-cIMT and energy intake (r = 0.196, *p*-value = 0.08), sugar intake (r = 0.194, *p*-value = 0.08), total protein intake (r = 0.187, *p*-value = 0.09), animal protein intake (r = 0.211, *p*-value = 0.06), and total lipid intake (r = 0.210, *p*-value = 0.06) (Supplementary Table S2).

Multiple linear regression analysis revealed that for every gram per kilogram per day-increase in SFA intake, max-cIMT was 0.046 mm higher (Supplementary Table S3). The intake of SFA was an independent predictor of max-cIMT [B (95% CI) = 0.046 (0.008–0.083), *p*-value = 0.018] after adjusting for gestational age at delivery, birth length, and birth weight. However, no significant interaction between max-cIMT and nutrient or energy intake was found when the group was considered (all *p*-values for interaction > 0.004 for multiple testing correction) (Supplementary Table S4). The estimated effect size for SFA intake levels revealed a statistical power of 73%.

### 3.4. Characterization of Nutritional Phenotypes Among ART Participants

K-means cluster analysis categorized ART participants into three distinct nutritional metabotypes based on their nutritional data (i.e., energy and macronutrient intakes). Significant nutritional differences were observed in energy, total carbohydrates, digestible polysaccharides, total proteins, vegetable proteins, total lipids, SFA, MUFA, PUFA, cholesterol, fiber, and sodium after adjusting for confounding factors between the four groups (comprising three established ART clusters and the control group) (Table 3). It is noteworthy that the mean values of energy and nutrient intake of participants belonging to cluster 2 closely resembled those of the control group. It is worth mentioning that participants in cluster 1 had the highest intake levels of energy, total carbohydrates, digestible polysaccharides, total proteins, vegetable proteins, total lipids, SFA, MUFA, PUFA, cholesterol, fiber, and sodium while participants in cluster 3 had the lowest intake values.

**Figure 2:**
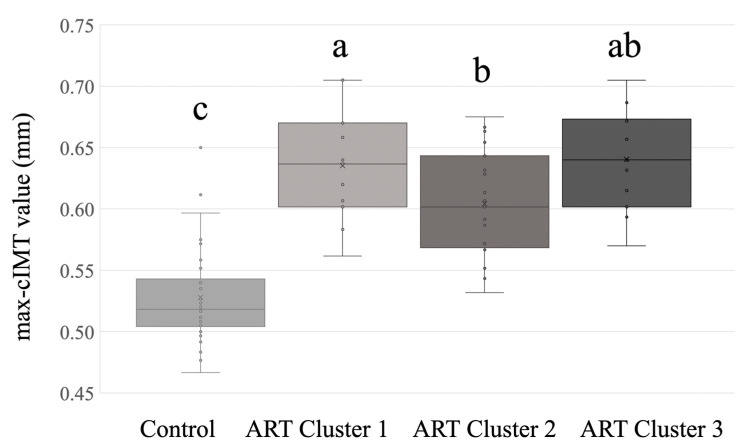
max-cIMT values among participants in the three ART clusters and the spontaneously conceived group. Data analyzed by ANCOVA adjusted for gestational age at delivery, sex, weight, and height. Means with superscripts without a common letter differ, *p*-value < 0.05 (Bonferroni post hoc test). ART, assisted reproductive technology. max-cIMT, carotid maximum intima–media thickness.

Differences in perinatal data and anthropometric characteristics between the nutritional metabotypes and the control group are presented in Supplementary Table S5. Cluster 2 ART children showed similar values to the control group and cluster 3 for weight and BMI percentile; however, these values were significantly higher than those of children in cluster 1. Interestingly, cluster 2 ART children exhibited lower max-cIMT values compared to children in clusters 1 and 3 (Supplementary Table S5 and Figure 2). However, the observed differences were specifically significant between ART Cluster 2 and ART Cluster 1 only when adjustments were made for covariates (Figure 2).

## 4. Discussion

This study explores whether the child’s nutritional intake could contribute to the observed increase in max-cIMT values in children conceived through ART [8,9].

Our study revealed notable differences in dietary intake between 3-year-old children conceived through ART and those conceived spontaneously. Specifically, ART children exhibited higher intake of total proteins, animal proteins and SFA, compared to their spontaneously conceived counterparts. However, our findings showed no association between SFA intake and max-cIMT in ART participants, although a positive association was observed in the entire participant sample (including ART and control subjects). To the best of our knowledge, this is the first study that assesses the dietary intake of ART children in comparison to a control group. Furthermore, no prior studies have explored the potential association between dietary intake and max-cIMT in ART subjects.

Our findings consistently align with previous evidence on SFA intake across life. However, the lack of standardization in the cIMT measurement protocol complicates direct comparisons. Studies vary in their approach, including, for example, the mean or maximum of single readings, the mean of multiple means, or the mean of the maximum values [11]. In our study, we used the maximum cIMT values as our primary variable. Similarly, a study in individuals aged 35–75 years indicated that each 10 g/day increase in SFA intake correlated with a 0.03 mm increment in the max-cIMT [22]. Other studies have reported improvements in cIMT after one year of reduced SFA intake in obese adolescents [23] and a significant reduction in atherosclerosis progression, as assessed by cIMT, in adults aged 55 years following a two-year nutritional intervention with decrease in SFA intake [24]. Moreover, a cohort from the Atherosclerosis Risk in Communities (ARIC) study [25] reported a positive association between cIMT values and SFA intake, assessed through a food frequency questionnaire in participants aged 45–64 years. Regarding PUFA intake, a beneficial effect on max-cIMT was observed [22]. Notably, this association between PUFA and cIMT did not show any effect in a five-year dietary supplement intervention among 8-year-old children [26], a result consistent with our findings. However, a significant positive association between cIMT and postnatal PUFA dietary intake in 4-year-old children was observed [13]. This discrepancy may be attributed to variations in dietary intake between children at early ages and newborns, exemplified by infant formulas and solid foods. Moreover, the lower consumption of nuts and fatty fish among children could be influenced by safety concerns related to choking risks, taste preferences, or limited exposure to seafood within their diet due to parental decisions.

One of the highlighted aspects of this work is emphasized by the identification of distinct dietary patterns among the ART subjects. Cluster 2, characterized by dietary patterns most similar to the control group, exhibited individuals with non-significant nutritional differences from the control group and showcased the lowest max-cIMT levels among all ART clusters. In contrast, the other two dietary patterns (Clusters 1 and 3) showed deviations from the control group and Cluster 2, involving either overeating and/or undereating, along with higher values of max-cIMT. Given the limited number of participants in Clusters 1 and 3, caution must be exercised in interpreting the results.

There is evidence suggesting that eating habits/patterns track from childhood to adulthood, and that unhealthy childhood nutrition, i.e., a diet poor in fruits and vegetables, can be linked to adulthood CVD risk factors, and to early vascular changes predicting the risk of CVD [12]. Our cohort of children exhibited dietary behaviors exceeding the recommended values of total fat and total protein intakes [15]. This finding aligns with evidence from the EsNuPi study [27] which analyzed a similar population of children aged one to under ten years old.

A study in children and adolescents with overweight and obesity indicated that adherence to healthy dietary pattern could prevent increased cIMT [28], while in hypercholesterolaemic children, a reduction in cIMT values was reported after 12 months of a Mediterranean diet intervention [29]. On the other hand, despite the significant associations between habitual dietary patterns and metabolic factors, no significant protective effect of the Mediterranean diet pattern was found against clinically silent carotid atherosclerosis—defined as increased cIMT and/or presence of carotid plaques [30]. Evidence from dietary patterns and cIMT values is overwhelmingly heterogeneous due to differences seen in dietary patterns based on location and culture, sample characteristics, and adjustment for confounders [31]. A gourd/root vegetable diet in the Bangladeshi population has been positively correlated with cIMT, while a balanced diet was associated with decreased cIMT [32]. On the other hand, the same study reported a positive association between animal protein diet and cIMT, but it was not statistically significant. These results are in accordance with our total population study since we also observed a positive correlation but with a trend of significance. However, evidence from protein intake is limited. High versus low protein intake in 5-year-old infancy was shown to have no influence on cIMT [33]. They speculated that subjects might have been too young to detect a significant change in cIMT in response to an early nutritional intervention.

The impact of ART on cardiovascular health remains inconclusive. Some studies have reported increased blood pressure and unfavorable changes in left ventricular structure and function in children conceived through ART aged 6 to 10 years, compared with children who were naturally conceived [3]. Several potential mechanisms that may explain the association of ART with abnormal left ventricular structure and dysfunction are that ART may be associated with increased risk of impaired lipid and glucose metabolisms, which is the pathophysiological basis of various cardiovascular diseases [4,34]. However, other studies have not found a significant increase in cardiovascular morbidity among ART individuals [35,36]. Given these discrepancies, further research with larger cohorts is needed to clarify cardiovascular risk in ART populations. Additionally, long-term cardiovascular risk is influenced by environmental factors acting through the processes of developmental plasticity and possibly epigenetic modifications [37]. ART itself may act as an early environmental factor during conception, potentially influencing perinatal outcomes and cardiovascular health due to increased oxidative stress levels. Reactive oxygen species (ROS) can be generated during the in vitro manipulations of gametes or embryos, as well as cryopreservation and culture media [38].

### Strengths and Limitations

Regarding the strengths of our study, this is the first study to examine the association between nutritional characteristics and their effect on max-cIMT in children conceived through ART. Moreover, the study design consists of a well-phenotyped cohort recruited from conception through childhood including complete infant and child carotid wall thickness data. Furthermore, the use of the k-means clustering method allows for the evaluation of dietary intake patterns instead of analyzing a specific nutrient. Some limitations should also be considered, such as the difficulty of working with the pediatric population and potential bias from reliance on parental recall for dietary intake data. We acknowledge that the cohort analyzed had no register about maternal and parental nutrition nor their dietary patterns in order to find a relation between that and max-cIMT. It is important to highlight that, previously, we did not find significant differences between the ART and spontaneously conceived groups in several potentially confounding factors, including maternal and parental smoking, early cardiovascular history, and socioeconomic status [8]. Future research should consider maternal and parental nutrition evaluation to provide a more comprehensive statistical analysis, along with genetic factors related to lipid metabolism or cardiovascular risk, to distinguish genetic from environmental contributions. Although the estimated effect size for SFA intake levels between groups demonstrated a statistical power of 73% in a limited sample size, it may still be insufficient to detect smaller but significant effects or to generalize the findings to other populations. Moreover, the cross-sectional design nested in a clinical trial did not allow for the assessment of causality between dietary intake and values of max-cIMT. A longitudinal study design would be valuable in future research to better clarify causal relationships. We also acknowledge that the changes reported here are subclinical, with most cardiovascular indexes lying within normal ranges. Although these differences are recognized as potential cardiovascular risk factors, their long-term persistence and association with adult cardiovascular function and disease remain to be proven.

## 5. Conclusions

In conclusion, our findings suggest that ART children exhibited a distinct dietary pattern characterized by higher consumption of total and animal proteins and SFA, compared to those conceived naturally.

While no specific nutrient intake among ART children was significantly associated with max-cIMT, SFA intake emerged as an independent predictor of max-cIMT within the entire study sample, including both ART and control subjects. Additionally, we categorized ART participants into three dietary patterns in this study. We observed that the pattern with a lower max-cIMT value closely mirrored the dietary habits of the control group. Further research is required to unravel the interindividual variations among subjects conceived through ART to formulate precise nutritional recommendations for personalized nutrition and preventive medicine.

## Figures and Tables

**Table 1:** Perinatal and current characteristics of the study groups.

Characteristics	Spontaneously Conceived (n = 42)	ART (n = 41)	*p*-Value
**Perinatal data**			
Females	24 (57.1)	19 (46.3)	0.33
Gestational age at delivery, weeks	40.1 ± 1.3	38.9 ± 1.5	<0.001
Birth weight, g	3338 ± 429	3136 ± 518	0.05
Birth weight percentile	54 ± 26	48 ± 32	0.17
Birth length, cm	50 ± 1	49 ± 2	0.010
Birth length percentile	52 ± 21	43 ± 31	0.036
Breastfeeding, %	24 (77.4)	26 (81.3)	0.71
Breastfeeding, months ^a^	4.5 ± 2.3	4.7 ± 1.6	0.37
**Characteristics at 3 years of age**			
Age at evaluation, years	3.2 ± 0.6	3.0 ± 0.5	0.13
**Child’s anthropometric data**			
Weight, kg	15.8 ± 1.9	15.1 ± 2.0	0.09
Weight percentile	59 ± 25	52 ± 30	0.10
Height, cm	98.4 ± 5.3	96.6 ± 5.2	0.14
Height percentile	59 ± 25	53 ± 29	0.22
BMI, kg/m^2^	16.4 ± 1.3	16.2 ± 1.7	0.55
BMI percentile	56 ± 27	48 ± 28	0.13
**Child’s vascular assessment**			
max-cIMT, mm	0.53 ± 0.04	0.62 ± 0.05	<0.001
Systolic blood pressure, mmHg	94.2 ± 10.5	94.6 ± 8.5	0.79
Diastolic blood pressure, mmHg	66.4 ± 10.6	65.4 ± 8.6	0.71

Data are mean ± standard deviation (SD) or n (%), as appropriate. ART, assisted reproductive techniques; BMI, body mass index; max-cIMT, carotid maximum intima–media thickness. ^a^ The calculation of the breastfeeding duration has been conducted only for individuals who were breastfed.

**Table 2:** Child’s dietary intake in the study participants.

Energy and Nutrient Intakes	Spontaneously Conceived (n = 42)	ART (n = 41)	*p*-Value	Adjusted *p*-Value ^a^
Energy, kcal/kg/day	82 ± 17	91 ± 21	0.044	0.10
Total carbohydrates, g/kg/day	9.1 ± 2.3	9.7 ± 2.7	0.24	0.51
Digestible polysaccharides, g/kg/day	4.8 ± 1.5	4.8 ± 1.7	0.95	0.68
Sugars, g/kg/day	4.3 ± 1.3	4.9 ± 1.5	0.022	0.07
Total proteins, g/kg/day	3.6 ± 0.9	4.1 ± 1.1	0.009	0.042
Animal proteins, g/kg/day	2.6 ± 0.9	3.1 ± 1.0	0.007	0.041
Vegetable proteins, g/kg/day	0.9 ± 0.3	1.0 ± 0.3	0.78	0.91
Total lipids, g/kg/day	3.5 ± 0.8	3.9 ± 1.0	0.06	0.07
SFA, g/kg/day	1.2 ± 0.3	1.4 ± 0.4	0.025	0.031
MUFA, g/kg/day	1.5 ± 0.4	1.6 ± 0.5	0.13	0.13
PUFA, g/kg/day	0.5 ± 0.2	0.5 ± 0.2	0.22	0.14
Cholesterol, mg/kg/day	13.0 ± 4.5	15.1 ± 5.7	0.07	0.19
Fiber, g/kg/day	0.7 ± 0.2	0.7 ± 0.3	0.89	0.92
Sodium, mg/kg/day	554 ± 160	645 ± 260	0.049	0.14

Data are mean ± standard deviation (SD). ART, assisted reproductive techniques; MUFA, monounsaturated fatty acids; PUFA, polyunsaturated fatty acids; SFA, saturated fatty acids. ^a^ ANCOVA test adjusted for gestational age at delivery, birth length and birth weight with the Bonferroni post hoc test, *p*-value < 0.05.

**Table 3:** Child’s energy and nutrient intakes of participants in the three ART clusters and the spontaneously conceived group.

Energy and Nutrient Intakes	Spontaneously Conceived (n = 42)	ART Cluster 1 (n = 11)	ART Cluster 2 (n = 19)	ART Cluster 3 (n = 11)	Adjusted *p*-Value
Energy, kcal/kg/day	82 ± 17 ^b^	119 ± 16 ^a^	85 ± 7 ^b^	73 ± 11 ^b^	<0.001
Total carbohydrates, g/kg/day	9.1 ± 2.3 ^b^	12.6 ± 2.9 ^a^	9.1 ± 1.4 ^ab^	8.0 ± 2.1 ^b^	0.004
Digestible polysaccharides, g/kg/day	4.8 ± 1.5 ^a^	6.4 ± 1.8 ^a^	4.7 ± 1.0 ^ab^	3.3 ± 0.9 ^b^	<0.001
Sugars, g/kg/day	4.3 ± 1.3	6.1 ± 1.5	4.4 ± 1.1	4.7 ± 1.3	0.078
Total proteins, g/kg/day	3.6 ± 0.9 ^b^	5.3 ± 1.3 ^a^	3.8 ± 0.5 ^ab^	3.4 ± 0.7 ^b^	0.006
Animal proteins, g/kg/day	2.6 ± 0.9	4.1 ± 1.4	2.8 ± 0.6	2.8 ± 0.6	0.17
Vegetable proteins, g/kg/day	0.9 ± 0.3 ^b^	1.2 ± 0.2 ^a^	1.0 ± 0.2 ^ab^	0.7 ± 0.1 ^c^	<0.001
Total lipids, g/kg/day	3.5 ± 0.8 ^b^	5.2 ± 0.7 ^a^	3.7 ± 0.5 ^b^	3.0 ± 0.5 ^b^	<0.001
SFA, g/kg/day	1.2 ± 0.3 ^b^	1.9 ± 0.3 ^a^	1.3 ± 0.3 ^b^	1.2 ± 0.3 ^b^	<0.001
MUFA, g/kg/day	1.5 ± 0.4 ^b^	2.2 ± 0.4 ^a^	1.5 ± 0.2 ^b^	1.2 ± 0.2 ^c^	<0.001
PUFA, g/kg/day	0.5 ± 0.2 ^bc^	0.7 ± 0.2 ^a^	0.5 ± 0.1 ^ab^	0.4 ± 0.1 ^c^	<0.001
Cholesterol, mg/kg/day	13.0 ± 4.5 ^b^	21.1 ± 6.1 ^a^	13.6 ± 3.7 ^ab^	11.8 ± 3.3 ^b^	0.020
Fiber, g/kg/day	0.7 ± 0.2 ^a^	0.9 ± 0.2 ^a^	0.8 ± 0.3 ^a^	0.5 ± 0.1 ^b^	<0.001
Sodium, mg/kg/day	554 ± 160 ^b^	820 ± 402 ^a^	603 ± 139 ^ab^	543 ± 157 ^b^	0.002

Data are mean ± standard deviation (SD). Means in a row with superscripts without a common letter differ (ANCOVA analysis adjusted for gestational age at delivery, sex, weight, and height with the Bonferroni post hoc test; *p*-value < 0.05). ART, assisted reproductive techniques; MUFA, monounsaturated fatty acids; PUFA, polyunsaturated fatty acids; SFA, saturated fatty acids.

## Data Availability

The datasets generated and analyzed during the current study are available from the corresponding authors upon reasonable request.

## Supplementary Materials

The following supporting information can be downloaded at https://www.mdpi.com/article/10.3390/nu17071189/s1: Supplementary Table S1, Children’s dietary intake and recommended dietary intake amounts in the entire participant sample (ART and control groups) (n = 83), in the control group (n = 42), and in the ART group (n = 41). Supplementary Table S2, Univariate regression analyses between nutrient intakes and max-cIMT in the entire participant sample (ART and control groups) (n = 83), in the control group (n = 42) and in the ART group (n = 41). Supplementary Table S3, Multivariate linear regression analyses for associations between nutrient intakes and max-cIMT in all participants (ART and control groups) (n = 83). Supplementary Table S4, Effect modification by group between nutrients and max-cIMT in all participants (ART and control groups) (n = 83). Supplementary Table S5: Characteristics of participants according to the control group and the three identified nutritional clusters of ART participants.

## Abbreviations

The following abbreviations are used in this manuscript:

3DFR	3-day food record
ANCOVA	Analysis of covariance
ANOVA	Analysis of variance
ART	Assisted reproductive technologies
BMI	Body mass index
cIMT	Carotid intima–media thickness
CVD	Cardiovascular disease
max-cIMT	Carotid maximum intima–media thickness
MUFA	Monounsaturated fatty acids
PUFA	Polyunsaturated fatty acids
RDA	Recommended Dietary Allowances
SFA	Saturated fatty acids
SD	Standard deviations
