# Supplementary material for: Influence of Dietary Intake on Carotid Maximum Intima–Media Thickness in Children Conceived Through Assisted Reproductive Techniques
Source: Nutrients. 2025 Mar 28;17(7):1189. doi: 10.3390/nu17071189 (PMC11990837; doi:10.3390/nu17071189)
Supplement: Supplementary file 1 [file nutrients-17-01189-s001.zip › nutrients-3517953-supplementary.pdf]

nutrients

*Supplementary Materials*

# Influence of Dietary Intake on Carotid Maximum Intima–Media Thickness in Children Conceived Through Assisted Reproductive Techniques

**Blanca Barrau-Martinez [1,2,†], Mireia Termes-Escalé [1,†], Brenda Valenzuela-Alcaraz [3], Rafael Llorach [1,2,4], Andreu Farran-Codina [1,2], Alba Tor-Roca [1,2,4], Eduard Gratacós [3,5], Fatima Crispi [3,5,*,‡] and Mireia Urpi-Sarda [1,2,4,*,‡]**

[1] Departament de Nutrició, Ciències dels Aliments i Gastronomia, Facultat de Farmàcia i Ciències de l'Alimentació, Campus de l'Alimentació de Torribera, Universitat de Barcelona (UB), 08921 Santa Coloma de Gramenet, Spain
blancabarrau@ub.edu (B.B.-M.); mireia.termes93@gmail.com (M.T.-E.); rafallorach@ub.edu (R.L.); afarran@ub.edu (A.F.-C.); albator@ub.edu (A.T.-R.)

[2] Institut de Recerca en Nutrició i Seguretat Alimentària (INSA-UB), Campus de l'Alimentació de Torribera, Universitat de Barcelona (UB), 08921 Santa Coloma de Gramenet, Spain

[3] Centre de Medicina Maternofetal i Neonatal de Barcelona (BCNatal), Hospital Clínic and Hospital Sant Joan de Déu, IDIBAPS, Universitat de Barcelona (UB), 08028, Barcelona, Spain
bren_val@hotmail.com (B.V.-A.); gratacos@clinic.cat (E.G.)

[4] Centro de Investigación Biomédica en Red de Fragilidad y Envejecimiento Saludable (CIBERFES), Instituto de Salud Carlos III, 28029 Madrid, Spain

[5] Centro de Investigación Biomédica en Red de Enfermedades Raras (CIBERER), Instituto de Salud Carlos III, 28029 Madrid, Spain

* Correspondence: fcrispi@clinic.cat (F.C.); murpi@ub.edu (M.U.-S.)

† These authors share first authorship.

‡ These authors share last authorship.

**Table S1.** Children's dietary intake and recommended dietary intake amounts in the entire participant sample (ART and control groups) (n=83), in the control group (n=42), and in the ART group (n=41).

| Energy or nutrient | Entire participant sample (n = 83) | RDA[a] to total sample (n = 83) | Control (n = 42) | RDA[a] to control (n = 42) | ART (n = 41) | RDA[a] to ART (n = 41) |
|---|---|---|---|---|---|---|
| Energy, kcal/kg/day | 86 ± 19 | 100 ± 5* | 82 ± 17 | 99 ± 5* | 91 ± 21 | 101 ± 4* |
| Total proteins, g/kg/day | 3.8 ± 1.02 | 1.2 ± 0.04* | 3.6 ± 0.9 | 1.2 ± 0.04* | 4.1 ± 1.1 | 1.2 ± 0.03* |
| Total lipids, % | 39 ± 4 | 30 ± 0* | 39 ± 4 | 30 ± 0* | 39 ± 4 | 30 ± 0* |
| Total carbohydrates, % | 43 ± 5 | 55 ± 0* | 44 ± 5 | 55 ± 0* | 43 ± 5 | 55 ± 0* |

Data are mean ± standard deviation (SD). ART, assisted reproductive techniques

*t-test, p-value < 0.001

[a] It was calculated from the Recommended Dietary Allowances (RDA) by National Research Council [1].

**Table S2.** Univariate regression analyses between nutrient intakes and max-cIMT in the entire participant sample (ART and control groups) (n=83), in the control group (n=42) and in the ART group (n=41).

| Energy or nutrient intake (kcal/kg/d or g/kg/d) | Entire participant sample (n = 83) | | Control (n = 42) | | ART (n = 41) | |
|---|---|---|---|---|---|---|
| | r[a] | p-value | r[a] | p-value | r[a] | p-value |
| Energy | 0.196 | 0.08 | 0.088 | 0.58 | 0.005 | 0.98 |
| Total carbohydrates | 0.123 | 0.27 | 0.047 | 0.78 | 0.029 | 0.86 |
| Digestible polysaccharides | 0.027 | 0.81 | 0.217 | 0.17 | -0.100 | 0.54 |
| Free sugar | 0.194 | 0.08 | -0.168 | 0.29 | 0.171 | 0.29 |
| Total protein | 0.187 | 0.09 | -0.003 | 0.98 | -0.088 | 0.58 |
| Animal protein | 0.211 | 0.06 | 0.037 | 0.82 | -0.092 | 0.57 |
| Vegetal proteins | 0.039 | 0.73 | 0.134 | 0.40 | -0.079 | 0.62 |
| Total lipids | 0.210 | 0.06 | 0.193 | 0.22 | -0.012 | 0.94 |
| SFA | 0.249 | **0.023** | 0.179 | 0.26 | 0.037 | 0.82 |
| MUFA | 0.179 | 0.10 | 0.156 | 0.32 | 0.014 | 0.93 |
| PUFA | 0.153 | 0.17 | 0.210 | 0.18 | -0.054 | 0.74 |
| Cholesterol | 0.117 | 0.29 | -0.004 | 0.98 | -0.101 | 0.53 |
| Fiber | 0.069 | 0.53 | 0.022 | 0.89 | 0.139 | 0.38 |
| Sodium | 0.256 | **0.019** | 0.272 | 0.08 | 0.034 | 0.83 |

[a] Pearson's correlation coefficient.

ART, assisted reproductive techniques; MUFA, monounsaturated fatty acids; PUFA, polyunsaturated fatty acids; SFA, saturated fatty acids.

**Table S3.** Multivariate linear regression analyses for associations between nutrient intakes and max-cIMT in all participants (ART and control groups) (n=83).

| Energy or nutrient intake (kcal/kg/d or g/kg/d) | B | CI 95% | p-value[a] |
|---|---|---|---|
| Energy | 0.001 | 0.000 – 0.001 | 0.09 |
| Total carbohydrates | 0.003 | -0.003 – 0.009 | 0.30 |
| Digestible polysaccharides | 0.000 | -0.008 – 0.009 | 0.95 |
| Free sugar | 0.010 | -0.001 – 0.020 | 0.06 |
| Total protein | 0.009 | -0.005 – 0.022 | 0.22 |
| Animal protein | 0.008 | -0.006 – 0.022 | 0.25 |
| Vegetal proteins | 0.012 | -0.042 – 0.066 | 0.65 |
| Total lipids | 0.014 | 0.000 – 0.029 | 0.05 |
| SFA | 0.046 | 0.008 – 0.083 | **0.018** |
| MUFA | 0.030 | -0.003 – 0.062 | 0.07 |
| PUFA | 0.073 | -0.014 – 0.159 | 0.10 |
| Cholesterol | 0.001 | -0.002 – 0.004 | 0.45 |
| Fiber | 0.028 | -0.027 – 0.084 | 0.31 |
| Sodium | 5.6 e-5 | -0.7 e-5 – 11.9 e-5 | 0.08 |

[a] Multivariate linear regression mode adjusted by gestational age at delivery, birth length and birth weight.

MUFA, monounsaturated fatty acids; PUFA, polyunsaturated fatty acids; SFA, saturated fatty acids.

**Table S4.** Effect modification by group between nutrients and max-cIMT in all participants (ART and control groups) (n=83).

| Energy or nutrient intake (kcal/kg/d or g/kg/d) | p-value for interaction[a] |
|---|---|
| Energy | 0.93 |
| Total carbohydrates | 0.75 |
| Digestible polysaccharides | 0.35 |
| Free sugar | 0.08 |
| Total protein | 0.72 |
| Animal protein | 0.68 |
| Vegetal proteins | 0.81 |
| Total lipids | 0.68 |
| SFA | 0.68 |
| MUFA | 0.84 |
| PUFA | 0.62 |
| Cholesterol | 0.63 |
| Fiber | 0.29 |
| Sodium | 0.35 |

[a] Multivariate linear regression analyses adjusted by gestational age at delivery, birth length and birth weight and case-control group.

MUFA, monounsaturated fatty acids; PUFA, polyunsaturated fatty acids; SFA, saturated fatty acids.

**Table S5.** Characteristics of participants according to the control group and the three identified nutritional clusters of ART participants.

| Characteristics | Control (n = 42) | ART Cluster 1 (n = 11) | ART Cluster 2 (n = 19) | ART Cluster 3 (n = 11) | *P*-value |
|---|---|---|---|---|---|
| **Perinatal data** | | | | | |
| Females | 24 (57.1) [ab] | 9 (81.8) [a] | 6 (31.6) [b] | 4 (36.4) [ab] | 0.035 |
| Gestational age at delivery, weeks | 40.1 ± 1.3 [a] | 39.4 ± 1.3 [ab] | 38.4 ± 1.6 [b] | 39.4 ± 1.4 [ab] | **<0.001** |
| Birth weight, g | 3338 ± 429 | 3041 ± 450 | 3118 ± 642 | 3263 ± 321 | 0.16 |
| Birth length, cm | 50 ± 1 | 49 ± 2 | 49 ± 2 | 50 ± 2 | **0.037** |
| Breastfeeding, % | 24 (77.4) | 5 (83.3) | 11 (73.3) | 10 (90.9) | 0.82 |
| Breastfeeding, months [a] | 4.5 ± 2.3 | 5.2 ± 1.1 | 4.2 ± 1.9 | 5.0 ± 1.3 | 0.50 |
| **Characteristics at 3 years of age** | | | | | |
| Age at evaluation, years | 3.2 ± 0.6 | 2.8 ± 0.7 | 3.2 ± 0.5 | 3.0 ± 0.1 | 0.08 |
| **Child's anthropometric data** | | | | | |
| Weight, kg | 15.8 ± 1.9 [a] | 13.4 ± 1.5 [c] | 15.9 ± 1.7 [a] | 15.7 ± 2.1 [ab] | **<0.001** |
| Height, cm | 98.4 ± 5.3 [a] | 93.8 ± 6.5 [b] | 98.8 ± 4.2 [ab] | 95.8 ± 4.2 [ab] | **0.023** |
| BMI, kg/m$^2$ | 16.4 ± 1.3 [ab] | 15.3 ± 1.5 [b] | 16.2 ± 1.3 [ab] | 17.0 ± 2.1 [a] | **0.044** |
| **Child's vascular assessment** | | | | | |
| max-cIMT, mm | 0.53 ± 0.04 [b] | 0.64 ± 0.05 [a] | 0.60 ± 0.04 [a] | 0.64 ± 0.04 [a] | **<0.001** |
| Systolic blood pressure, mmHg | 94.2 ± 10.5 | 93.2 ± 8.5 | 94.1 ± 7.8 | 96.8 ± 10.2 | 0.84 |
| Diastolic blood pressure, mmHg | 66.4 ± 10.6 | 64.7 ± 9.9 | 64.4 ± 8.0 | 67.8 ± 8.6 | 0.79 |

Data are mean ± standard deviation (SD) or n (%), as appropriate. Means in a row with superscripts without a common letter differ (one-factor analysis of variance (ANOVA) with the Bonferroni post hoc test. p-value < 0.05). ART, assisted reproductive techniques; BMI, body mass index; max-cIMT, carotid maximum intima-media thickness.

[a] The calculation of breastfeeding duration has been conducted for individuals who received breastfeeding.

## References

1. National Research Council (US) Subcommittee on the Tenth Edition of the Recommended Dietary Allowances. Recommended Dietary Allowances: 10th Edition; National Academies Press (US), 1989; ISBN 0309046335.
